# Treatment beyond progression with anti-PD-1/PD-L1 based regimens in advanced solid tumors: a systematic review

**DOI:** 10.1186/s12885-021-08165-0

**Published:** 2021-04-17

**Authors:** Francesco Spagnolo, Andrea Boutros, Federica Cecchi, Elena Croce, Enrica Teresa Tanda, Paola Queirolo

**Affiliations:** 1 Medical Oncology 2, IRCCS Ospedale Policlinico San Martino, Largo R. Benzi 10, 16132 Genoa, Italy; 2 Melanoma, Sarcoma & Rare Tumors Division, European Institute of Oncology (IEO), Milan, Italy

**Keywords:** Melanoma, Immunotherapy, Immune-related response criteria, Treatment beyond progression, Anti-PD-1

## Abstract

**Background:**

Treatment beyond progression with immunotherapy may be appropriate in selected patients based on the potential for late responses. The aim of this systematic review was to explore the impact of treatment beyond progression in patients receiving an anti-PD-1/PD-L1 based regimen for an advanced solid tumor.

**Methods:**

A systematic literature search was performed to identify prospective clinical trials reporting data on overall response rate by immune-related criteria and/or the number of patients treated beyond conventional criteria-defined PD and/or the number of patients achieving a clinical benefit after an initial PD with regimens including an anti-PD-1/PD-L1 agent which received the FDA approval for the treatment of an advanced solid tumor.

**Results:**

254 (4.6%) responses after an initial RECIST-defined progressive disease were observed among 5588 patients, based on 35 trials included in our analysis reporting this information. The overall rate of patients receiving treatment beyond progressive disease was 30.2%, based on data on 5334 patients enrolled in 36 trials, and the rate of patients who achieved an unconventional response among those treated beyond progressive disease was 19.7% (based on 25 trials for a total of 853 patients).

**Conclusion:**

The results of our systematic review support the clinical relevance of unconventional responses to anti-PD-1/PD-L1-based regimens; however, most publications provided only partial information regarding immune-related clinical activity, or did not provide any information at all, highlighting the need of a more comprehensive report of such data in trials investigating immunotherapy for the treatment of patients with advanced tumors.

## Précis

Immunotherapy treatment beyond progression (TBP) may be appropriate in selected patients due to the potential of late responses. In the studies included in our analysis, 30% of patients received TBP and the overall rate of late responses was 4.6%.

## Background

Patients with advanced solid tumors who are treated with immunotherapy may develop atypical patterns of response, which initially meet conventional response criteria for progressive disease (PD) but later result in tumor regression or prolonged disease stabilization (SD) [1, 2]. To evaluate the peculiar antitumor effects of immunotherapy, a number of immune-related response criteria were developed. As a general principle, by these criteria, the initial increase in tumor burden or the appearance of new lesions is not assessed as PD until confirmation at a subsequent tumor assessment, providing that patients clinical conditions are not deteriorating [1, 3, 4]. Therefore, in selected patients, treatment beyond progression with immunotherapy may be appropriate based on the potential for late responses [5–10].

To assess the rate of atypical responses (i.e. tumor regressions or prolonged disease stabilization after RECIST-defined PD) in patients with advanced solid tumors who received anti-PD-1 immunotherapy, in 2017 we reviewed the results of 38 clinical trials for a total of 7069 patients [2]. In summary, the proportion of patients treated beyond progression ranged from 11 to40%; atypical responses were evaluated in 19 clinical trials and 151 atypical responses were observed among 2400 patients, for an overall rate of atypical responses of 6% [2].

Since then, anti-PD-1 and PD-L1 drugs have been integrated into standard-of-care across many cancer types and many indications. Notably, anti-PD-1/PD-L1 agents also became the keystone of new combinations with chemotherapy, targeted therapy and other immunotherapies, and new clinical trials with anti-PD-1/PD-L1 based regimens have been increasing exponentially.

In view of the uncertainty regarding whether discontinuation of immunotherapy, based on conventional response criteria, may be premature for at least a subset of patients who could derive a late benefit from treatment continuation, most clinical trials of immunotherapies allow for treatment beyond RECIST-defined PD [2]. However, the clinical benefit of treatment beyond progression remains difficult to assess, and whether atypical responses are observed also in regimens including chemotherapy, targeted therapy and other immunotherapies is not clear.

In light of the considerably high number of studies and new combinations, the aim of this systematic review was to update our previously published analysis [2] to further explore the impact of atypical responses and treatment beyond progression in patients receiving an anti-PD-1/PD-L1 based regimen for an advanced solid tumor, and to assess if atypical patterns of response were also observed in patients treated with new combination regimens.

## Methods

**Fig. 1:**
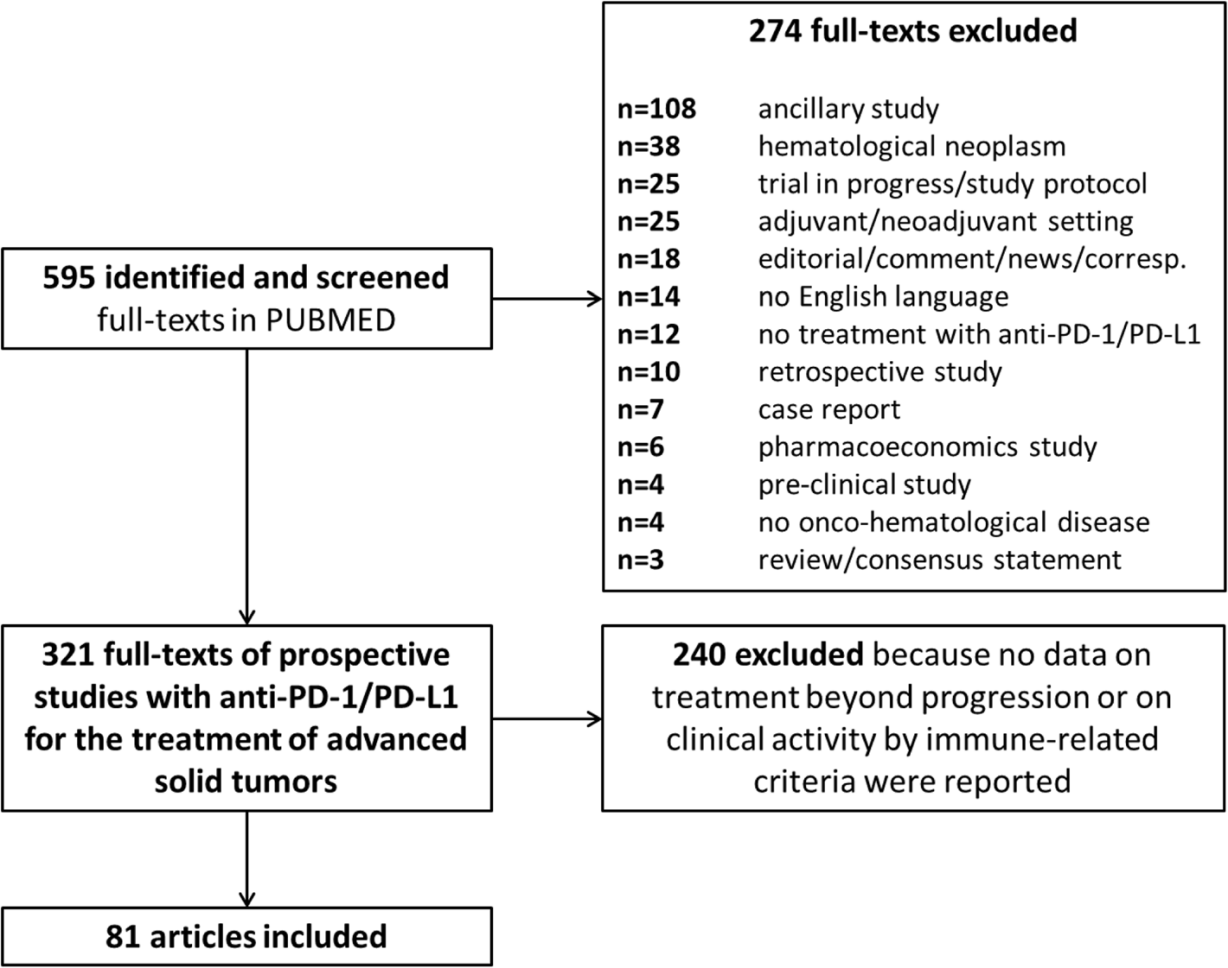
The PRISMA flowchart summarizing the process for the identification of the eligible studies

Preferred Reporting Items for Systematic Reviews and Meta-Analyses (PRISMA) guidelines were used for the conduct and reporting of this systematic review (Fig. 1) [11].

Prospective clinical trials reporting data on overall response rate (ORR) by immune-related criteria and/or the number of patients treated beyond conventional criteria-defined PD and/or the number of patients achieving a clinical benefit after an initial PD during treatment with regimens including an anti-PD-1/PD-L1 which received the FDA approval for the treatment of an advanced solid tumor were included in this systematic review. The following information was extracted from each report: name of study/study code, ClinicalTrials.gov Identifier, first author and date of publication, study design, treatment regimen, type of cancer, number of patients evaluated for response, time to first imaging, ORR by conventional response evaluation criteria, ORR by immune-related response criteria, response rate after initial PD, treatment beyond progression rate. Only data for arms including an anti-PD-1/PD-L1 agent were reported in our review. Supplementary material was also reviewed when available. Abstracts and conference papers were not included in our review. We also excluded other reviews, meta-analyses and retrospective analyses of case series. In the event that a study was published in multiple articles, the most recent data were extracted and reported in the tables.

The definition of atypical response slightly varied across studies, but always followed the principles of the immune-related response criteria proposed by Wolchock et al. in 2009 (i.e. tumor regressions or prolonged disease stabilization after RECIST-defined PD) [1].

Clinical trials were identified by a computerized search of the PubMed data-base with the string (“nivolumab”[MeSH Terms] OR “nivolumab”[All Fields] OR “nivolumab s”[All Fields] OR “pembrolizumab”[Supplementary Concept] OR “pembrolizumab”[All Fields] OR “cemiplimab”[Supplementary Concept] OR “cemiplimab”[All Fields] OR “atezolizumab”[Supplementary Concept] OR “atezolizumab”[All Fields] OR “durvalumab”[Supplementary Concept] OR “durvalumab”[All Fields] OR “avelumab”[Supplementary Concept] OR “avelumab”[All Fields]) and the filter for article type “Clinical Trial”. The search was performed on the 24th July 2020.

Data were independently extracted by two investigators (F.S. and A.B.) to ensure homogeneity of collection and to rule out the effect of subjectivity in data gathering and entry. Disagreements were resolved by iteration, discussion and consensus.

Only descriptive statistics were conducted to obtain a pooled response rate by immune-related response criteria and a pooled rate of patients treated beyond progression for each group of studies (anti-PD-1/PD-L1 as single agents or in combination with chemotherapy, targeted therapy and other immunotherapy).

## Results

Among 321 full-texts of prospective studies investigating anti-PD-1/PD-L1 drugs for the treatment of advanced solid tumors, 240 articles were excluded because they did not met the key inclusion criteria of our systematic analysis (i.e. they did not report data on ORR by immune-related criteria and/or on the number of patients treated beyond conventional criteria-defined PD and/or on the number of patients achieving a clinical benefit after an initial PD during treatment with regimens including an anti-PD-1/PD-L1 agent); therefore, only 81 articles were considered eligible and were included in the analysis (Fig. 1), for a total of 9644 patients who received an anti-PD-1/PD-L1-based regimen for an advanced solid tumor, and for whom data on immune-related clinical activity was reported.

The results of the studies included in our systematic review are summarized according to treatment regimen (anti-PD-1/PD-L1 as single agent, anti-PD-1/PD-L1 in combination with other immunotherapies, anti-PD-1/PD-L1 in combination with targeted therapy, anti-PD-1/PD-L1 in combination with chemotherapy) in Tables 1, 2, 3 and 4.

**Table 1:** Summary of results of clinical trials with anti-PD-1/PD-L1 as single agents (only data for anti-PD-1/PD-L1 arms are reported)

Study name/code (NCT)	Study phase	Type of Cancer	Treatment	Patients evaluated for response	Time to first tumor assessment (weeks)	ORR by RECIST criteria	ORR by immune-related response criteria	Rate of patients treated beyond progression	ORR after initial PD	First author and date of publication
**15–286** **(NCT02673333)**	2	Adrenocortical carcinoma	Pembrolizumab	39	9	9 (23.1%)	Not reported	Not reported	2 (5.1%)	Raj 2020 [12]
**16–032** **(NCT02730130)**	2	Breast cancer	Pembrolizumab plus RT	17	13	3 (17.6%)	3 (17.6%)	Not reported	Not reported	Ho 2019 [13]
**2014–1315** **(NCT02364076)**	2	Thymic carcinoma	Pembrolizumab	40	6	9 (22.5%)	Not reported	Not reported	0 (0%)	Giaccone 2018 [14]
**20,151,049** **(NCT02658019)**	2	Hepatocellular carcinoma	Pembrolizumab	28	9	9 (32.1%)	Not reported	15 (53.6%)	1 (3.6%)	Feun 2019 [15]
**Alliance A091401** **(NCT02500797)**	2	Sarcoma	Nivolumab	38	6	2 (5.3%)	Not reported	18 (47.4%)	0 (0%)	D’Angelo 2018 [16]
**Attraction-2** **(NCT02267343)**	3	Gastric cancer	Nivolumab	268	6	30 (11.2%)	Not reported	95 (35.5%)	Not reported	Kang 2017 [17]
**CD-ON-MEDI4736–1108** **(NCT01693562)**	1/2	UC	Durvalumab	42	6	13 (31.0%)	Not reported	2 (4.8%)	2 (4.8%)	Massard 2016 [18]
		NSCLC		256		39 (15.2%)		99 (38.7%)	Not reported	Antonia 2019 [19]
**CA-210-001** **(NCT00729664)**	1	Advanced solid tumors	Nivolumab	160	6	17 (10.6%)	Not reported	Not reported	4 (2.5%)	Brahmer 2012 [20]
**CheckMate-003** **(NCT00730639)**	1	RCC	Nivolumab	34	8	10 (29.4%)	Not reported	Not reported	3 (8.8%)	MCDermott 2015 [21]
		Melanoma		107		33 (30.8%)	Not reported	Not reported	4 (3.7%)	Topalian 2014 [22]
		NSCLC		129		22 (17.1%)	Not reported	Not reported	6 (4.7%)	Gettinger 2015 [23]
**CheckMate-004** **(NCT01024231)**	1	Melanoma	Nivolumab	30	8	6 (20.0%)	Not reported	Not reported	3 (10.0%)	Wolchock 2013 [24]
**CheckMate-010** **(NCT01354431)**	2	RCC	Nivolumab	168	6	35 (20.8%)	38 (22.8%)	36 (21.4%)	2 (1.2%)^a^	Motzer 2015 [25], George 2016 [8], Pignon 2019 [6]
**CheckMate-012** **(NCT01454102)**	1	NSCLC	Nivolumab	52	11	12 (23.1%)	Not reported	Not reported	3 (5.8%)	Gettinger 2016 [26]
**CheckMate-017** **(NCT01642004)**	3	Squamous NSCLC	Nivolumab	135	9	27 (20.0%)	Not reported	27 (20.0%)	9 (6.7%)	Brahmer 2015 [27]
**CheckMate-025** **(NCT01668784)**	3	RCC	Nivolumab	406	8	Not reported	Not reported	153 (37.7%)	20 (4.9%)	Escudier 2017 [28]
**CheckMate-026** **(NCT02041533)**	3	NSCLC	Nivolumab	211	6	55 (26.1%)	Not reported	77 (36.5%)	Not reported	Carbone 2017 [29]
**CheckMate 032** **(NCT01928394)**	1/2	SCLC	Nivolumab	98	6	10 (9.8%)	Not reported	30 (30.6%)	Not reported	Antonia 2016 [30]
		UC		78		19 (24.4%)	Not reported	31 (39.7%)	9 (11.5%)	Sharma 2016 [31]
**CheckMate-037** **(NCT01721746)**	3	Melanoma	Nivolumab	120	9	38 (31.7%)	Not reported	37 (30.8%)	10 (8.3%)	Weber 2015 [32]
**CheckMate-057** **(NCT01673867)**	3	Non-squamous NSCLC	Nivolumab	292	9	56 (19.2%)	Not reported	71 (24.3%)	16 (5.5%)	Borghaei 2015 [33]
**CheckMate-063** **(NCT01721759)**	2	Squamous NSCLC	Nivolumab	117	8	17 (14.5%)	Not reported	22 (18.8%)	4 (3.4%)	Rizvi 2015 [34]
**CheckMate-066** **(NCT01721772)**	3	Melanoma	Nivolumab	210	9	84 (40.0%)	Not reported	54 (25.7%)	17 (8.1%)	Robert 2015 [35]
**CheckMate-067** **(NCT01844505)**	3	Melanoma	Nivolumab	316	12	140 (44.3%)	Not reported	97 (30.7%)	Not reported	Wolchok 2017 [36]
**CheckMate-141** **(NCT02105636)**	3	HNSCC	Nivolumab	240	9	Not reported	Not reported	62 (25.8%)	3 (1.3%)^b^	Haddad 2019 [7]
**CheckMate-275** **(NCT02387996)**	2	UC	Nivolumab	265	8	52 (19.6%)	Not reported	70 (26%)	24 (9.1%)	Sharma 2017 [37]
**FIR** **(NCT01846416)**	2	NSCLC	Atezolizumab	137	6	30 (21.9%)	32 (23.4%)	Not reported	2 (1.5%)	Spigel 2018 [38]
**IMvigor210** **(NCT02108652)**	2	Urothelial cancer	Atezolizumab	310	9	45 (15%)	58 (19%)	121 (39%)	21 (6.8%)	Rosenberg 2016 [39]
**IND 121564** **(NCT02085070)**	2	Melanoma	Pembrolizumab	18	8	4 (22.2%)	Not reported	1 (5.6%)	Not reported	Goldberg 2016 [40]
		NSCLC		18		6 (33.3%)		1 (5.6%)	2 (11.1%)	
**Javelin Merkel 200** **(NCT02155647)**	2	Merkel cell carcinoma	Avelumab	88	6	28 (31.8%)	Not reported	Not reported	1 (1.1%)	Kaufman 2016 [41]
**Javelin Solid Tumor** **(NCT01772004)**	1b	Adrenocortical	Avelumab	50	6	3 (6.0%)	3 (6.0%)	Not reported	1 (2.0%)	Le Tourneau [42]
		NSCLC		184		22 (12.0%)	22 (12.0%)	Not reported	0 (0%)	Gulley 2017 [43]
		Breast cancer		168		5 (2.9%)	Not reported	Not reported	2 (1.2%)	Dirix 2018 [44]
		Ovarian cancer		125		12 (9.6%)	16 (12.8%)	Not reported	7 (5.6%)	Disis 2019 [45]
		UC		161		27 (16.8%)	28 (17.4%)	Not reported	1 (0.6%)	Patel 2018 [46]
**JAVELIN Solid Tumor JPN trial** **(NCT01943461)**	1	Advanced solid tumors	Avelumab	40 (dose- expansion cohort)	6	4 (10.0%)	4 (10.0%)	Not reported	Not reported	Doi 2019 [47]
**KEYNOTE-001** **(NCT01295827)**	1	NSCLC	Pembrolizumab	550	12	121 (24.4%)^c^	145 (26.4%)	Not reported	Not reported	Garon 2015 [48], Garon 2019 [49]
		Melanoma		327		Not reported	Not reported	Not reported	24 (7.3%)	Hodi 2016 [9]
				581		194 (33.4%)	260 (44.8%)	Not reported	Not reported	Ribas 2016 [50]
**KEYNOTE-002** **(NCT01704287)**	2	Melanoma	Pembrolizumab	361	12	84 (23.3%)	Not reported	72 (20.0%)	Not reported	Ribas 2015 [51]
**KEYNOTE-011** **(NCT01840579)**	1	Multiple tumor types	Pembrolizumab	9	6	2 (22.2%)	2 (22.2%)	1 (11.1%)	1 (11.1%)	Shimizu 2016 [52]
**KEYNOTE-012** **(NCT01848834)**	1b	UC	Pembrolizumab	27	8	7 (25.9%)	Not reported	Not reported	0 (0.0%)	Plimack 2017 [53]
		HNSCC		56		12 (21.4%)	Not reported	Not reported	1 (1.8%)	Seiwert 2016 [54]
**KEYNOTE-016** **(NCT01876511)**	2	CRC, MMR–deficient cancers	Pembrolizumab	35	12	9 (25.7%)	9 (25.7%)	Not reported	0 (0.0%)	Le 2015 [55]
**KEYNOTE-224** **(NCT02702414)**	2	Hepatocellular carcinoma	Pembrolizumab	104	9	18 (17.3%)	18 (17.3%)	Not reported	Not reported	Zhu 2018 [56]
**NCI-2016-00545** **(NCT02721732)**	2	Multiple rare cancers	Pembrolizumab	110	9	Not reported	15 (13.6%)	34 (30.9%)	6 (5.5%)	Naing 2020 [57]
**OAK** **(NCT02008227)**	3	NSCLC	Atezolizumab	425	6	58 (13.7%)	68 (16.0%)	168 (39.5%)	Not reported	Gandara 2018 [10], von Pawel 2019 [58]
**ONO-4538-25** **(NCT02582125)**	2	NSCLC	Nivolumab	53	6	5 (9.4%)	Not reported	17 (32.1%)	Not reported	Chen 2020 [59]
**ONO-4538-07** **(Not available)**	2	Esophageal squamous-cell carcinoma	Nivolumab	64	6	14 (21.9%)	16 (25.0%)	Not reported	2 (3.1%)	Kudo 2017 [60]
**NivoMes** **(NCT02497508)**	2	Mesothelioma	Nivolumab	34	6	9 (26.5%)	Not reported	Not reported	3 (8.8%)	Quispel-Janssen 2018 [61]
**PCD4989g** **(NCT01375842)**	1	Urothelial bladder cancer	Atezolizumab	65	6	17 (26.2%)	Not reported	Not reported	1 (1.5%)	Powles 2014 [62]
		RCC		62		9 (14.5%)	Not reported	28 (45.2%)	6 (9.7%)	McDermott 2016 [63]
		HNSCC		32		7 (21.9%)	Not reported	10 (31.3%)	Not reported	Colevas 2018 [64]
		Breast		115		11 (9.6%)	15 (13.0%)	Not reported	3 (2.6%)	Emens 2019 [65]
**R2810-ONC-1540** **(NCT02760498)**	2	Cutaneous squamous cell carcinoma	Cemiplimab	78	8	41 (52.6%)	Not reported	Not reported	2 (2.6%)	Migden 2020 [66]
**SARC028** **(NCT02301039)**	2	Sarcoma	Pembrolizumab	80	8	9 (11.3%)	10 (12.5%)	Not reported	3 (3.8%)	Tawbi 2017 [67]
**Umin 000005714** **(Not available)**	2	Ovarian cancer	Nivolumab	20	8	3 (15.0%)	Not reported	Not reported	1 (5.0%)	Hamanishi [68]

Abbreviations: *CRC* colorectal cancer; *HNSCC* head and neck squamous cell carcinoma, *MMR* mismatch repair, *NCT* ClinicalTrials.gov Identifier, *NSCLC* non-small-cell lung carcinoma, *ORR* overall response rate, *PD* progressive disease, *RCC* renal-cell carcinoma, *RT* radiotherapy, *SCLC* small-cell lung carcinoma, *UC* urothelial cancer

^a^additional 12 patients achieved < 30% tumor reduction

^b^overall, 25 patients achieved < 30% tumor reduction

^c^based on centrally-assessed RECIST 1.1 data reported by Garon et al. 2015 on 495 patients

**Table 2:** Summary of results of clinical trials with combinations of anti-PD-1/PD-L1 with immunotherapy

Study name/code (NCT)	Study phase	Type of Cancer	Treatment	Patients evaluated for response	Time to first tumor assessment (weeks)	ORR by RECIST 1.1 criteria	ORR by immune-related response criteria	Patients treated beyond progression	Response rate after initial PD	First author and date of publication
**102,323** **(NCT02523469)**	1b	NSCLC	Nivolumab plus ALT-803 [IL-15 superagonist]	21	6	6 (28.6%)	Not reported	Not reported	1 (4.8%)	Wrangle 2018 [69]
**ABC** **(NCT02374242)**	2	Melanoma	Nivolumab plus ipilimumab	35	12	16–17 (46–57%)^a^	Not reported	21 (60.0%)	4 (11.4%)	Long 2018 [70]
**Alliance A091401** **(NCT02500797)**	2	Sarcoma	Nivolumab plus ipilimumab	38	6	6 (15.8%)	Not reported	8 (21.1%)	1 (2.6%)	D’Angelo 2018 [16]
**CheckMate-004** **(NCT01024231)**	1	Melanoma	Nivolumab plus ipilimumab	52	8	21 (40.4%)	Not reported	Not reported	4 (7.7%)	Wolchock 2013 [24]
**CheckMate 032** **(NCT01928394)**	1/2	Small-cell lung carcinoma	Nivolumab plus ipilimumab	118	6	25 (21.2%)	Not reported	21 (17.8%)	Not reported	Antonia 2016 [30]
**CheckMate-067** **(NCT01844505)**	3	Melanoma	Nivolumab plus ipilimumab	314	12	183 (58.3%)	Not reported	62 (19.8%)	Not reported	Wolchok 2017 [36]
**D4190C00006** **(NCT02000947)**	1b	NSCLC	Durvalumab plus tremelimumab	63	8	11 (17.5%)	Not reported	Not reported	2 (3.2%)	Antonia 2016 [71]
**J1L-AM-JZGA** **(NCT02009449)**	1b	Advanced solid tumours	Pegilodecakin plus anti-PD-1	96	8	Not reported	29 (30.2%)	Not reported	Not reported	Naing 2019 [72]
**NIBIT-MESO-1** **(NCT02588131)**	2	Mesothelioma	Durvalumab plus tremelimumab	40	12	Not reported	11 (27.5%)	13 (32.5%)	1 (2.5%)	Calabrò 2018 [73]

Abbreviations: *NCT* ClinicalTrials.gov Identifier, *NSCLC* non-small-cell lung carcinoma, *ORR* overall response rate, *PD* progressive disease

^a^ Intracranial and extracranial response rate, respectively

**Table 3:** Summary of results of clinical trials with combinations of anti-PD-1/PD-L1 with targeted therapy

Study name/code (NCT)	Study phase	Type of Cancer	Treatment	Patients evaluated for response	Time to first tumor assessment (weeks)	ORR by RECIST 1.1 criteria	ORR by immune-related response criteria	Patients treated beyond progression	Response rate after initial PD	First author and date of publication
**20,150,932** **(NCT02636725)**	2	Sarcoma	Pembrolizumab plus axitinib	32	12	8 (25.0%)	8 (25.0%)	4 (12.5%)	Not reported	Wilky 2019 [74]
**A4061079** **(NCT02133742)**	1b	RCC	Pembrolizumab plus axitinib	52	12	38 (73.1%)	Not reported	8 (15.4%)	1 (1.9%)	Atkins 2018 [75]
**BTCRC-GU14–003** **(NCT02348008)**	1b/2	RCC	Pembrolizuamb plus bevacizumab	58	6	33 (56.9%)	Not reported	7 (12.1%)	Not reported	Dudek 2020 [76]
**CheckMate-012** **(NCT01454102)**	1	NSCLC	Nivolumab Plus erlotinib	20	11	3 (15.0%)	Not reported	Not reported	1 (5.0%)	Gettinger 2018 [77]
**GP28328** **(NCT01633970)**	1b	RCC	Atezolizumab plus bevacizumab	10	6	4 (40.0%)	Not reported	2 (20.0%)	1 (10.0%)	Wallin 2016 [78]
**KEYNOTE-146** **(NCT02501096)**	1b/2	Endometrial cancer	Pembrolizumab plus lenvatinib	108	6	44 (40.7%)	47 (43.5%)	Not reported	Not reported	Makker 2020 [79]

Abbreviations: *NCT* ClinicalTrials.gov Identifier, *NSCLC* non-small-cell lung carcinoma, *ORR* overall response rate, *PD* progressive disease, *RCC* renal-cell carcinoma

**Table 4:** Summary of results of clinical trials with combinations of anti-PD-1/PD-L1 with chemotherapy

Study name/code (NCT)	Study phase	Type of Cancer	Treatment	Patients evaluated for response	Time to first tumor assessment (weeks)	ORR by RECIST 1.1 criteria	ORR by immune-related response criteria	Patients treated beyond progression	Response rate after initial PD	First author and date of publication
**CheckMate-012** **(NCT01454102)**	1	NSCLC	Nivolumab plus standard chemotherapy	56	10^a^	24 (42.9%)	Not reported	Not reported	1 (1.8%)	Rizvi 2016 [80]
**GP28328** **(NCT01633970)**	1b	Breast cancer	Atezolizumab plus chemotherapy	33	8	13 (39.4%)	Not reported	6 (18.2%)	3 (9.1%)	Adams 2019 [81]
**PembroPlus** **(NCT02331251)**	1b/2	Pancreatic adenocarcinoma	Pembrolizumab plus gemcitabine and nab-paclitaxel	15	Not reported	3 (20.0%)	3 (20.0%)^a^	Not reported	0 (0.0%)^b^	Weiss 2017 [82]
**NCI-2015-01310** **(NCT02538510)**	2	HNSCC and salivary gland cancer	Pembrolizumab plus vorinostat	50	9	12 (24.0%)	Not reported	12 (24.0%)	1 (2.0%)^c^	Rodriguez 2019 [83]
**PEMBROSARC** **(NCT02406781)**	2	Sarcomas	Pembrolizumab plus CP	50	6	0 (0.0%)	1 (2.0%)	Not reported	1 (2.0%)	Toulmonde 2018 [84]

Abbreviations: *CP* cyclophosphamide, *HNSCC* head and neck squamous cell carcinoma, *NCT* ClinicalTrials.gov Identifier, *NSCLC* non-small-cell lung carcinoma, *ORR* overall response rate, *PD* progressive disease, *SD* stable disease

^a^ Per the original study protocol, tumor response was first assessed at week 6. However, due to the chance of early pseudoprogression at this time point, the protocol was amended to perform the first tumor assessment at week 10

^b^2 patients achieved immune-related SD after RECIST-defined PD

^c^an additional patient achieved > 6 months SD

Forty-four studies investigating anti-PD-1/PD-L1 as single agents were included in our analysis, for a total of 8383 patients (Table 1) [6–10, 12–68]. The number of responses achieved after an initial conventional criteria-defined PD was reported in 35 studies, for a total of 5053 patients evaluated for response; a response after an initial PD was achieved by 232 patients (4.6%). The rate of patients who received treatment with anti-PD-1/PD-L1 as single agents beyond RECIST-defined PD was 31.8%, based on 26 trials reporting this information for a total of 4554 patients. In 18 studies reporting both the number of patients treated beyond PD and the number of responses achieved after initial PD, among 783 subjects who received anti-PD-1/PD-L1 treatment beyond PD, 156 patients (19.9%) achieved a response after initial RECIST-defined PD. Finally, in 13 studies both the ORR according to conventional and immune-related criteria were reported, and a total of 549 and 674 responses were observed, respectively. Responses after initial RECIST-defined PD were observed across multiple tumor types and varied slightly across the tumor types more represented in our analysis. Specifically, the pooled rate of responses after initial RECIST-defined PD was 4.0% for lung cancer, 6.1% for urothelial cancer, 7.2% for melanoma and 4.6% for renal-cell carcinoma.

Nine studies of clinical trials with immunotherapy combination regimens including an anti-PD-1/PD-L1 agent were included in our systematic review, for a total of 777 patients (Table 2) [16, 24, 30, 36, 69–73]. In 6 trials reporting response rate after initial PD, 13/249 patients (5.2%) achieved a response after initial RECIST-defined PD. The rate of patients treated beyond PD was 22.9%, based on 5 studies reporting this information for a total of 545 patients. In 3 trials reporting both the number of patients treated beyond PD and the number of responses achieved after initial PD, among 42 subjects who received an anti-PD-1/PD-L1-based immunotherapy combination treatment beyond PD, 6 patients (14.3%) achieved a response after an initial PD. No combination immunotherapy trials reported both the ORR according to conventional and immune-related criteria.

Six studies investigating treatment with anti-PD-1/PD-L1 in combination with targeted agents were included in the analysis, for a total of 280 patients (Table 3) [74–79]. Three responses (3.7%) after initial PD were observed in 3 trials reporting this information; 21/152 patients (13.8%) were treated beyond PD. Only in 2 trials both response rate after initial PD and rate of patients treated beyond PD were reported, with 2/10 subjects (20%) achieving response after initial RECIST-defined PD. In the 2 trials reporting both RECIST-defined ORR and ORR by immune-related criteria, 52 and 55 patients achieved a response, respectively.

In the 5 trials of anti-PD-1/PD-L1 in combination with chemotherapy, 6 (2.9%) responses after PD were achieved in a total of 204 patients evaluated for response (Table 4) [80–84]. Based on 2 studies reporting this information, 18/83 patients (21.7%) were treated beyond PD, and among these 18 patients, 4 (22.2%) achieved a response after an initial PD.

Overall, based on 35 trials included in our analysis reporting data on unconventional responses, 254 responses after an initial RECIST-defined PD were observed among 5588 patients, for an overall rate of 4.6%. The overall rate of patients receiving treatment beyond PD was 30.2% based on 36 trials (5334 patients), and the overall rate of patients who achieved a response after initial RECIST-defined PD among those treated beyond PD was 19.7% (25 trials, 853 patients). Finally, in 17 trials (2800 patients) reporting ORR by both conventional and immune-related criteria, 604 and 733 responses were achieved, respectively.

## Discussion

In a pooled analysis of individual patient data made by the US Food and Drug Administration (FDA) in 2018, all submissions of trial reports and data in support of marketing applications for anti-PD-1 drugs as single agent or in combination with other drugs for the treatment of patients with advanced melanoma that allowed for continuation of treatment beyond RECIST-defined PD were analyzed to investigate the effect of treatment beyond PD and to define which subset of patients derive benefit from extended treatment. The finding of this study showed that among the 8 multicenter clinical trials included in the review for a total of 2624 patients receiving immunotherapy, 692/1361 patients (51%) received anti-PD-1 treatment beyond RECIST-defined PD, and 95/500 evaluable patients (19%) had a response after initial RECIST-defined PD, representing 14% of the 692 patients treated beyond PD and 4% of all 2624 patients treated with immunotherapy [5]. Based on these results, the authors concluded that treatment beyond PD could not be recommended because the clinical benefit remained to be proven, but that might be appropriate for selected patients identified by specific criteria at the time of progression [5].

In our current systematic review, we found an overall rate of 4.6% of responses after initial RECIST-defined PD, similar to that reported by the individual patient data pooled analysis made by the FDA in patients with melanoma (4%) [5]. In our analysis, we found that responses after initial RECIST-defined PD may be achieved across multiple tumor types and multiple treatment regimens based on anti-PD-1/PD-L1 agents, including combinations with targeted therapy, chemotherapy and other immunotherapy. Our results suggested that the impact of immunotherapy treatment beyond RECIST-defined PD is similar regardless of treatment regimens. Notably, the overall rate of patients treated beyond PD achieving a subsequent response was also similar to that reported by the FDA in advanced melanoma (19.7 and 19%, respectively) [5]. The pooled rate of response after RECIST-defined PD was higher for melanoma (7.2%) than lung cancer (4.0%), which may reflect the higher conventional RECIST-defined response rate observed for anti-PD-1/PD-L1 treatment as single agent in melanoma patients as compared as patients with lung cancer.

Despite treatment beyond RECIST-defined PD was allowed in the vast majority of clinical trials analyzed during our literature search (> 90%; data not shown), we found that data on treatment beyond PD and immune-related anti-tumor clinical activity was largely under-reported, with only 81 articles meeting our inclusion criteria among 321 prospective trials full-texts analyzed, for a rate of 25%. In addition to that, partial results were often reported, with only a fraction of articles reporting data on both the rate of patients treated beyond PD and those who achieved a response after initial RECIST-defined PD, representing a limitation of our analysis. Other limitations of our study are the heterogeneity of the included studies in terms of design, populations, treatment regimens, time to first tumor assessment and response evaluation, and the small sample size for the groups of patients treated with combination treatments. Moreover, the impact of treatment beyond PD may have been underestimated because long-lasting disease stabilizations were not included. Some pseudoprogressions may be associated to an early imaging (i.e. 4–6 weeks); nevertheless, in the studies included in our analysis, time to first tumor assessment was never lower than 6 weeks, ranging from 6 to 12 weeks (with the exception of one study with a very small sample size, where first evaluation was performed at 13 weeks), and responses after RECIST-defined PD were observed regardless of time to first tumor assessment.

Despite these limitations, the results of our systematic review highlight the clinical relevance of responses to anti-PD-1/PD-L1-based regimens after initial RECIST-defined PD, and support further investigation into the development of tools that may assist clinicians for the selection of patients who may derive a benefit from extended immunotherapy treatment beyond RECIST-defined PD. Circulating tumor DNA has emerged as a promising blood-based biomarker for monitoring disease status of patients with advanced cancers, and may play an important predictive role into differentiating pseudo-progressions from true progressions, as observed in a cohort of 125 patients with advanced melanoma who were treated with anti-PD-1 antibodies [85].

Immune-related response criteria were developed to facilitate consistent trial design and data collection; however, most publications provided only partial information regarding immune-related clinical activity, or did not provide any information at all, despite the option of treatment beyond PD and response evaluation by immune-related criteria being mentioned in the study protocols, highlighting the need of a more comprehensive report of such data in trials investigating immunotherapy for the treatment of patients with advanced tumors.

## Data Availability

All data generated or analysed during this study are included in this published article.

## Abbreviations

ORR: Overall response rate
PD: Progressive disease
SD: Disease stabilization
